# Prostate Cancer: Emerging Modifiable Risk Factors and Therapeutic Strategies in the Management of Advanced Cancer

**DOI:** 10.3390/life14091094

**Published:** 2024-08-30

**Authors:** Sabrina Bossio, Lidia Urlandini, Anna Perri, Francesco Conforti, Antonio Aversa, Silvia Di Agostino, Vittoria Rago

**Affiliations:** 1 Department of Experimental and Clinical Medicine, Magna Graecia University of Catanzaro, 88100 Catanzaro, Italy; sabrina.bossio@unicz.it (S.B.); aversa@unicz.it (A.A.); 2 Department of Pharmacy, Health and Nutritional Sciences, University of Calabria, 87036 Rende, Italy; lidiaurlandini@gmail.com (L.U.); vittoria.rago@unical.it (V.R.); 3 Pathology Unit, Annunziata Hospital, 87100 Cosenza, Italy; francesco.conforti@unical.it; 4 Department of Health Sciences, Magna Graecia University of Catanzaro, 88100 Catanzaro, Italy; sdiagostino@unicz.it

**Keywords:** prostate cancer (PCa), unhealthy lifestyle, environmental exposure, heavy metals, persistent organic pollutants, advanced prostate cancer, treatment

## Abstract

Prostate cancer (PCa) is the third highest cause of cancer death in men. PCa is a very heterogeneous tumor form in terms of grade, phenotypes, and genetics, often accompanied by complex networks. PCa is characterized by slow growth that does not compromise the patient’s quality of life, unlike more aggressive forms showing rapid growth and progression. Early diagnosis, even for the most aggressive forms, increases the possibilities of cure with less aggressive treatments and fewer side effects. However, it is important to know how to decrease the exposure to modifiable risk factors, including diet, sedentary life, smoking and alcohol, can represent an effective tool to reduce the incidence of PCa. In addition, the chronic exposure to environmental factors, most of which act as endocrine disruptors, is the focus of recent studies for their potential role in promoting the onset and progression of PCa. Although molecular therapies and clinical trials for biomarker identification have been introduced into the management of PCa, these still lag behind research performed in other solid tumors. This review provides an overview of the modifiable factors of PCa, linked to lifestyle and environmental pollutants, which together with the development of new therapeutic targets, can reduce the incidence of PCa and improve the quality of life of patients.

## 1. Introduction

Prostate Cancer (PCa) is the most frequent solid tumor affecting the world’s male population [1]. Despite the progress in the early detection of PCa and its treatment over the years, this cancer remains a deadly disease. However, if action is taken promptly, the risk of the outcome being fatal is low [2]. PCa is a fairly heterogeneous disease both genetically and epidemiologically, and knowing the non-modifiable and modifiable risk factors of disease is crucial to direct primary and secondary prevention [3]. It is well-established that there are three major non-modifiable risk factors of PCa, and these are aging, race, and family history [4]. A positive family history of PCa, which is strongly related to the genetic background, is certainly one of the most important risk factors of PCa, and it is directly dependent on the degree of kinship, the number of affected relatives, and the degree of malignancy and mortality. Furthermore, a family history of high-grade or metastatic PCa increases the risk in descendants of developing a more aggressive and fatal form of PCa [5,6]. The physiological process of cellular senescence has a role in the onset and progression of many aging-related diseases, including cancer. This phenomenon is associated with the secretome produced by senescent cells, which includes pro-inflammatory and pro-oxidant factors, collectively referred to as the senescence-associated secretory phenotype that can promote tumorigenesis and tumor metastasis [7]. During aging, the physiological loss of glandular homeostasis favors the development of a pro-inflammatory and pro-fibrotic prostatic microenvironmental suitable for prostate cancer growth and progression [8,9]. In addition to non-modifiable risk factors, evidence suggests that modifiable risk factors may play a critical role in PCa development and progression. Most of these factors are part of unhealthy habits, particularly spreading among young people, such as a diet rich in fats and sugar, a sedentary lifestyle, alcohol abuse, and smoking. A growing body of evidence reports that these unhealthy habits, promoting a low chronic pro-oxidant and inflammatory status, can favor cancer development, highlighting that a potential primary strategy to counteract the spread of many cancers is encouragement to live a healthy lifestyle [10]. In the scenario of modifiable risk factors of cancer, and specifically of endocrine neoplasia including prostate cancer, many studies assessed the tumorigenic properties of endocrine-disrupting chemicals (EDC), such as pesticides, phthalates, heavy metals, and particulate matter, showing the highest cancer risk after EDC exposure [11].

In the field of PCa management, another issue is that many androgen-deprivation-therapy (ADT)-treated patients develop castration-resistant PCa (CRPC). It is well known that although ADT is initially effective, resistance subsequently develops, the AR signaling remains active in the absence of androgens, and the development of CRPC is often associated with the presence of metastases. Despite several therapeutic advancements, metastatic CRPC (mCRPC) has a poor prognosis with a survival rate below 2–3 years [12]. Therefore, in addition to counteracting PCa onset and progression through primary prevention, ongoing studies aimed to develop new therapeutic approaches to overcome castration resistance and improve survival rates.

Here, we examined the current evidence on the impact of unhealthy habits and environmental pollution on PCa development, and the progress that has been made in the treatment of advanced prostatic cancer.

## 2. Materials and Methods

The current state of knowledge reported in this narrative review was conducted using PubMed and Google Scholar. We selected original articles (in vitro, in vivo, and human studies), and reviews in the English language using the search terms: “Prostate Cancer AND Risk factors”; “Cancer AND diet”; “Prostate Cancer AND Obesity”; “Prostate Cancer AND unhealthy behaviours”; “Androgen Deprivation Therapy AND Prostate Cancer”; “Prostate Cancer AND Pollution”; “Prostate Cancer AND heavy metals”; “Prostate Cancer AND Persistent Organic Pollutants”; “Endocrine Disruptors AND Endocrine cancers”; “Prostate Tumor Microenvironment”; “Preclinical models AND Prostate Cancer”; “Prostate Cancer treatment”; “CRCP treatment”; “New drugs AND metastatic Prostate Cancer”. The selection of each study was based on the title and abstract; after critical evaluation of the the full text, it was included in the review.

## 3. Prostate Cancer and an Unhealthy Lifestyle

In recent years, a scientific consensus has been reached on the evidence that most types of cancers, as well as non-communicable diseases, are preventable through the adoption of a healthy lifestyle and that unhealthy habits increase the risk of cancer [13]. Many lines of evidence suggest that the risk of PCa could be reduced by improving one’s lifestyle. Furthermore, the recent 20-year follow-up study of Plym et al. showed that among men with an increased genetic risk, premature deaths may be preventable by implementing healthy lifestyle choices [14].

Regarding modifiable risk factors of PCa, evidence suggests that diet, smoking, excessive alcohol consumption, and physical inactivity may play a role in prostate tumorigenesis. Although the role of these factors has not been completely elucidated yet, it is presumable that their interaction increases the risk of PCa development [15]. Furthermore, emerging studies pointed out the urgent need to evaluate modifiable environmental risk factors of PCa, such as cadmium, arsenic, and air pollution as potential targets for cancer prevention [16].

### 3.1. Diet

**Figure 1:**
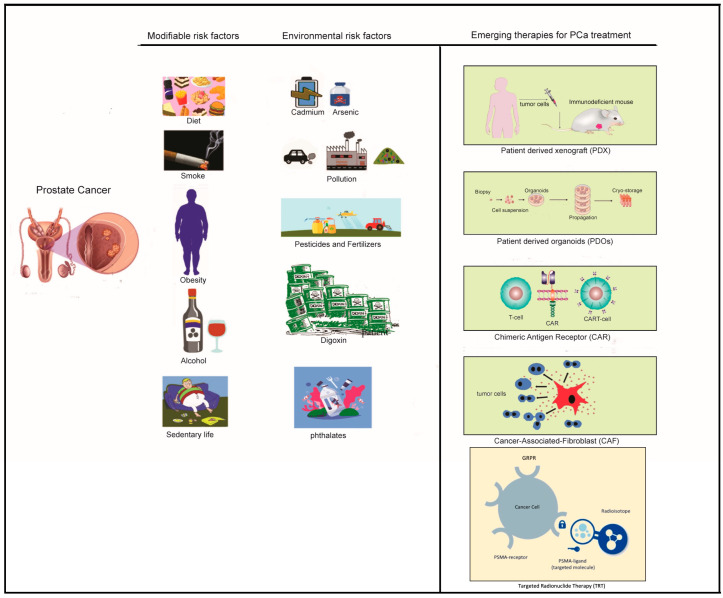
Schematic representation of the main modifiable risk factors and environmental contaminants involved in the onset and progression of prostate cancer (**left** panel) and emerging therapeutic strategies in the management of advanced prostate cancer (**right** panel). Figure created at https://www.freepik.com/ (accessed on 22 July 2024).

Many bodies of evidence demonstrate that a balanced diet is crucial for a healthy and long life [17]. Some literature focused on the association between PCa and nutritional factors, such as fat, carbohydrates, proteins, vitamins, and polyphenols, demonstrating that they may have a pathogenetic role in the onset and progression of PCa. However, contrasting results have emerged from clinical studies, and even now, it is unclear which nutrients have a protective or detrimental effect on PCa incidence and progression [18]. Data arising from animal and clinical studies evaluated the role of dietary patterns on the risk of developing PCa. Interesting findings showed that the Western dietary pattern is positively correlated with the risk of PCa and that adherence to a healthy Mediterranean diet has a protective effect against the risk of PCa [15,18,19,20,21]. The detrimental effects promoted by the Western diet are attributed to the high content of calories, fats, and oxidizing agents that promote overall intraprostatic inflammation that favors PCa growth [20]. In addition, the foods of the Western diet have a high glycemic index, which increases the risk of cancer by causing hyperinsulinemia [22]. Concerning this topic, the recent clinical study by Fu and collegues reported that hyperinsulinemia and inflammation may be potential mechanisms linking dietary patterns with the risk of aggressive PCa, suggesting that avoiding inflammatory and hyperinsulinemic patterns may be a useful tool to prevent clinically relevant PCa [23]. To further strengthen the role of inflammation and hyperinsulinemia in PCa progression, the wide longitudinal observational study CaPSURE conducted among men with non-metastatic PCa, suggests that diets with higher inflammatory and insulinemic potential were associated with a higher risk of PCa progression [24]. An emerging topic also related to diet patterns is the role of microbiota in cancer development. Despite the numerous studies published on the association between gut microbiota and colon cancer, few studies have investigated the relationship between gut microbiota and PCa. The available experimental studies have shown that the gut microbiome affects the growth and progression of PCa through metabolites derived from gut microbes, suggesting the existence of a “gut–prostate axis” [25]. Liss et al. analyzed the fecal microbiome in men who underwent a prostate biopsy, finding a great diversity between men with PCa compared to those without PCa, suggesting that a particular set of bacteria and their related metabolic pathways can contribute to PCa development [26]. A study comparing the microbiota of children in Burkina Faso, who have a high-fiber diet, with those of European children who consume a conventional Western diet, reported that the microbiota of children from Burkina Faso contained significantly less enterobacteriaceae and higher levels of short-chain fatty acids, such as butyric acid, which exerts anti-tumor actions through the induction of apoptosis and proliferation restriction [25] (Table 1, Figure 1).

### 3.2. Smoking

Cigarette smoking, tobacco combustion, and smoke inhalation, including passive smoking, are among the leading causes of cancer death, and recently their role in PCa has also been investigated [27]. The data emerging from the literature, including meta-analyses, still do not clarify the role of smoking in PCa development. However, according to a recent study that analyzed a cohort of 17 studies, it appears that cigarette smoking has an inverse association with PCa. Thus, in smokers, the incidence of developing PCa is lower, but patients who are affected by PCa have greater resistance to treatment, and the risk of death due to PCa is higher [28,29]. Other meta-analyses show that cigarette smoking alone has a low incidence of PCa, and this seems to be more widely reflected in other research works and data comparisons, but it has also been seen that when associated with other risk factors such as alcohol, the incidence undergoes a significant increase [30,31] (Table 1, Figure 1).

### 3.3. Alcohol

Excessive alcohol intake is certainly a risk factor for the development and proliferation of various forms of cancer, but not in PCa [32]. In fact, from the data collected, it seems that there is no correlation between alcohol intake and the development of PCa or an increased risk of mortality. The studies carried out so far have led to inconsistent results and need further investigation [33] (Table 1, Figure 1).

### 3.4. Sedentary Lifestyle

Recent studies and meta-analyses have shown a 30% risk reduction in PCa mortality in patients with an active lifestyle. Further studies show short-term clinical benefits in patients who have switched from a sedentary lifestyle to a more active one by incorporating even light physical activity into their daily routine. In patients who engage in occupational or competitive physical activity, the risk of developing PCa is significantly reduced [34]. Certainly, the data collected shows that men with PCa who led an active lifestyle or started exercising after diagnosis could delay or even avoid treatments and their side effects, as well as maintain a better quality of life [35]. Quality of sleep is an important aspect of having a healthy and balanced lifestyle. Some studies have investigated the correlation between sleep and PCa, and from the results, there seems to be no correlation between sleep duration and the risk of developing PCa [36]. On the other hand, there was a remarkable association between sleep quality and PCa risk; patients whose sleep quality was very poor were more likely to develop more aggressive forms of PCa [37]. Patients with PCa who engaged in frequent physical activity and who had a healthy lifestyle had more stable telomeres and reduced PSA levels compared to sedentary patients [38] (Table 1, Figure 1).

Collectively, the data emerging from the literature suggests that unhealthy habits can act as modifiable risk factors for cancer development, including PCa. Therefore, although the adoption of healthy behavior is not sufficient to prevent the onset of PCa, as many factors are implicated in its pathogenesis, it is evident that following a healthy lifestyle does not expone to the systemic detrimental effects promoted by the low pro-oxidant and pro-inflammation status induced by unhealthy habits.

**Table 1:** Unhealthy lifestyle, environmental factors, and PCa development.

Unhealthy Lifestyle	Effects on PCa Onset and Progression		References
**Diet**	ROS production, Inflammation, Hyperinsulinemia, Gut microbiome disrupted		[18,19,20,21,22,23,24,25,26]
**Smoking**	High resistance to treatment		[28,29,30,31]
**Alcohol**	No correlation with PCa		[32,33]
**Sedentary life**	Active lifestyle reduces the risk of mortality. Poor sleep quality induces aggressive forms of PCa		[34,35,36,37,38]
**Environmental Factors**	**Effects on PCa onset and progression**	**Mechanisms of action**	**References**
**Heavy metals**	Tumor progression	Increased levels of MMP-9; DNA metylation; K-RAS activation; apoptosis inhibition SUMOylation of AR	[39,40,41,42,43,44]
**Persistent Organic Pollutants**	Increase in aggressiveness and metastasis progression	Activation/inhibition of transcriptional activity of target genes by AHR-XRE activation; up-regulation of ACAT1 by AHR activation; Up-regulation of MMP-9 exerted by AHR	[45,46]
**Environmental carcinogens**	Excessive production of ROS drives the onset of PCa	Cellular damage, changes DNA sequence, proteins alterations	[47,48]

## 4. Prostate Cancer and Environmental Factors

Some researchers have studied environmental pollution for their potential pathogenetic role in promoting the onset and progression of PCa. Many chemicals, whose sources of exposure are diverse and vary around the world, can act as endocrine-disrupting chemicals (EDCs). These exogenous substances interfere with any aspect of hormone action and can disrupt hormonal homeostasis. Generally, EDCs can act as agonist and antagonist hormonal receptors, blocking or activating intracellular signal transduction pathways. Lifelong exposure to EDCs can lead to a cumulative and/or synergic effect that can increase the incidence of hormone-related neoplasia, including PCa [11].

### 4.1. Heavy Metals

Cadmium (Cd) and Arsenic (As) are the main heavy metals that act as EDCs and may be implicated in prostate tumorigenesis. Cd, a transition metal, and As, a metalloid, can affect several molecular mechanisms that lead to PCa onset. Both Cd and As are naturally present in the Earth’s crust, but they are also found in the natural environment due to the development of industrial activities. There are epidemiological studies that reported the correlation between high exposure to As in drinking water and high incidence of PCa in various regions of the USA [49,50]. The biological mechanisms linking As exposure to an increased risk of PCA appear to involve epigenetic processes. These mechanisms enhance cell proliferation and survival and reduce apoptotic events [49].

Additionally, exposure to As promotes androgen independence, a condition commonly linked to advanced stages of PCA and a poor prognosis because of resistance to certain treatments [51]. Several in vitro studies have demonstrated that low-level exposure to As in human prostate stem cells induces transformation in cancer stem-like cells characterized by an increased release of matrix metalloproteinase-9 (MMP-9), inhibition of apoptosis, DNA methylation, and activation of K-RAS oncogene [39,40,41]. An increased expression of anti-apoptotic genes BCL2 and BCL-XL, which induced resistance to cell death, has also been observed, while the expression of MMP-9 supported the metastatic process [42]. Furthermore, during the development of PCa, normal cells undergo a process where tumor suppressor genes are silenced due to promoter methylation. This silencing is associated with heightened activity of the DNMT3b DNA methyltransferase enzyme [43]. In addition, the arsenic-induced malignant transformation of PCa is driven by K-RAS upregulation along with the proliferation by the RAS/ERK pathway [40].

Similarly, in vitro studies have demonstrated that Cd exposure induces malignant transformation of non-tumorigenic and normal human prostate epithelial cells through abnormal DNA methylation and elevated MMP-9 secretion, resulting in increased aggressiveness and tumor progression [52]. In addition, interesting data showed that Cd can act as a steroid hormone, binding with high affinity to the hormone-binding domain of the androgen receptor (AR), which plays an important role in prostate carcinogenesis [53]. Epidemiological data and animal and in vitro studies suggested that Cd is a potential prostate carcinogenic agent. A population-based prospective cohort study supported the role of dietary Cd exposure in PCa development [54]. Wu and collegues described that Cd treatment on prostate cancer cell lines acts on the hormone-binding domain of AR, which plays an important role in the transcriptional process of prostate carcinogenesis [44]. They demonstrated that Cd regulates the SUMOylation, the conjugation of small ubiquitin-related modifier (SUMO) proteins of AR, involved in post-translational changes that regulate several physiological processes. The SUMOylation observed after Cd treatment consisted of a major AR transcriptional activity with downregulation of AR SUMOylation [44].

### 4.2. Persistent Organic Pollutants (POPs)

Compounds that are associated with the risk of PCa are the persistent organic pollutants (POPs), such as polychloribiphenyls (PCBs), dioxin, and pesticides, which are defined by the Stockholm Convention as chemicals that accumulate in living tissue exerting a toxic effect due to their resistance to degradation. Excessive exposure to these organic pollutants is documented to induce an increase in aggressiveness and metastasis progression of PCa by several studies. Even if the exact mechanism of action of dioxin and PCBs is unknown in PCa, dioxin and PCBs exert their effects through the aryl hydrocarbon receptor (AHR) that binds to the xenobiotic response element (XRE) to activate or inhibit transcriptional activity of specific target genes. Therefore, it seems that these organic compounds could be stored in periprostatic adipose tissue probably due to their lipophilic nature [45]. Several studies show that dioxin and PCBs could have pro- or anti-proliferative activity in PCa cell lines, but also in other tumors in correlation to the methodology applied, type of cell lines, and timing of exposure of dioxin/PCBs used [55]. Buñay et al. reported that PCa progression has involved the enzymes acetyl-coenzyme A (CoA) acetyltransferase-1 (ACAT1), which sustain ATP production via reversible reaction during lipogenesis and ketogenesis processes. They found that the increase of invasion and accelerated cell growth and metastasis was correlated to an up-regulation of ACAT1 by AHR activation [45]. In 2016, the effects of dioxin and PCBs on PCa were tested by using an animal model called “Transgenic Adenocarcinoma of the Mouse Prostate (TRAMP)”. The authors found that dioxin and PCBs exerted pro- or anti-carcinogenic effects in correlation to the time to exposure but also to the nature of the AHR ligand [56].

Therefore, the link between dioxin/PCBs and AHR ligand is an important connection, because when dioxin/PCB is bound to AHR, it induces an AHR nuclear translocation and, after a dimerization process by AHR nuclear translocator (ARNT), it leads to the transcription of AHR target genes. This mechanism is made principally by enzymes of cytochrome P450 that are involved in the process of detoxing from environmental pollutants [57]. In addition, it has been demonstrated that in animal models, dioxin/PCBs exposure induces the up-regulation of MMP-9, previously described in human prostate cancer cell lines, in which the production of MMP-9 was stimulated by AHR in the dose and in time-dependent mode [46].

Recently, Alwadi and colleagues described the effects of phenols and parabens, products with endocrine-disrupting properties, as risk factors of PCa in a study conducted in a U.S. men’s cohort. Phenols and parabens act as agonists or antagonists of sex hormones, and their exposure on humans can be due to ingestion of contaminated food or inhalation or absorption through the skin [58]. They found that the higher levels of phenols/parabens metabolites in urine samples were associated with an increased risk of PCa in the male population cohort who frequently consumed fast food. Additionally, software analysis using Gene Ontology (GO) and Kyoto Encyclopedia of Genes and Genomes (KEGG) pathways identified high-level genes in the aggressive stages of PCa tissue, indicating their potential role in carcinogenesis [58].

Interestingly, several studies have reported that exposure to environmental carcinogens can stimulate oxidative stress, causing damage to tumors, increasing the expression of proto-oncogenes, and inducing the malignant transformation of cells [47]. Usually, reactive oxygen species (ROS), whether generated by environmental pollution or other endogenous/exogenous factors, are neutralized by antioxidant enzyme systems. However, the excessive production of ROS could generate cellular damage, changes in the DNA structure, and alteration of proteins, inducing events driving the onset of PCa [48] (Table 1).

In summary, experimental in vitro and in vivo evidence suggests that environmental pollution could contribute to the risk of PCa development and progression, occurring through various mechanisms, including inflammation, oxidative stress, and genetic damage. However, to date, the underlying mechanisms have not been completely elucidated, and only a few clinical studies can support the link between environment and PCa. Undoubtedly, reducing exposure to harmful pollutants through regulatory measures and personal actions is essential for lowering cancer risk and improving public health.

## 5. Preclinical Models and Emerging Therapies in the Treatment of Advanced Prostate Cancer

Localized PCa can be effectively treated with radical prostatectomy and radiotherapy, while metastatic prostate cancer often remains incurable and fatal. In advanced PCa, ADT is the standard treatment. However, inevitable CRPC has limited treatment options with a low survival rate [59]. Recent studies have identified that genetic abnormalities are related to PCa and its progression to CRPC, such as mutations in AR, TP53, PTEN, and BRCA1/2 genes. These findings support the development of new experimental therapies [60]. Research on PCa is often based on in vitro studies of human prostatic cancer cells such as LNCaP, DU-145, and PC-3, used to uncover the molecular mechanisms driving progression and treatment resistance. While these cell lines help identify predictors of treatment response and resistance, they fail to capture the full diversity and heterogeneity of human prostate cancer. The growing knowledge of the molecular mechanisms through which modifiabfle risk factors can contribute to prostate cancerogenesis, including the role of tumor microenvironmental, allowed the development of new therapeutic approaches, mainly to prevent the spread of PCa. Therefore, the research is moving beyond the traditional 2D cell lines to patient-derived models that better reflect the complexity of the tumor ecosystem [60].

### 5.1. Preclinical Models of PCa

A new research tool in PCa studies is the patient-derived xenograft (PDX) model. This model involves transplanting tumor tissue directly from a patient into an immunodeficient mouse or other animal. The tumor grows in the animal, maintaining characteristics of the original patient’s cancer, thus providing a more accurate representation of the tumor’s biology and the advantage of reproducing the complexity of the tumor microenvironment (TME), resulting in a useful tool that can serve as a clinical predictive model for personalized therapeutic strategies. Despite their advantages, PDX models have limitations, such as the lack of a fully functional human immune system in the host animal, which can affect the accuracy of studies on immune-related therapies. In addition, it has been observed that PDX in humanized mice causes a rapid loss of human stromal components of implanted tumor tissue, which are replaced by mouse TME during engraftment [61]. Preclinical studies used the PDX model to identify and validate biomarkers associated with PCa. Several studies have reported the ability to predict treatment response, disease progression, or prognosis by using the molecular and genetic characteristics of PDX tumors with patient data [60]. Recently, novel PDX models have been characterized. These include hormone-naïve, androgen-sensitive, and CRPC, as well as neuroendocrine prostate cancer (NEPC), a rare form of PCa that can develop either de novo or as a resistance to therapies [62]. In this biobank, it was possible to identify alteration in androgen signaling, DNA repair, and PI3K pathways by genomic analysis, and the responses to androgen deprivation, PARP inhibitors, and chemotherapy treatment [62] (Table 2, Figure 1) were evaluated. A further emerging tool in PCa research is the use of patient-derived organoids (PDOs). These 3D cultures are developed from patient tissues and retain the genetic and histological characteristics of the original tumors, making them valuable for studying disease mechanisms and testing drug responses. PDOs are typically cultured using a basement membrane extract like matrigel, which supports cell growth and differentiation. This approach has enabled the development of organoids that mirror the molecular diversity and clinical heterogeneity of PCa, including various genetic mutations and AR expression levels [63,64]. One of the key advantages of using PDOs in PCa research is their ability to mimic the tumor microenvironment, which is crucial for understanding tumor–stroma interactions and the impact of different cell types on tumor growth and drug response. For instance, co-culturing prostate cancer organoids with stromal cells has been shown to improve their survival rate and formation efficiency, providing a more accurate model of in vivo conditions [65]. PDOs have been particularly useful in studying CRPC and NEPC, both of which are challenging to treat due to their aggressive nature and resistance to conventional therapies. PDO models of these subtypes have helped researchers investigate the underlying mechanisms of therapeutic resistance and identify potential new treatment strategies [60,64]. Overall, PDOs are proving to be a robust platform for precision medicine in PCa, offering a more personalized approach to understanding and treating this complex disease (Table 2, Figure 1).

### 5.2. Novel Putative Therapies in the Management of Advanced PCa

The emergence of novel therapies has prolonged the life expectancy of metastatic CRPC patients. The availability of tools to predict and monitor therapy response is crucial to personalize the treatment, changing it early in the resistance phase when necessary. In recent years, a new promising therapeutic strategy seems to be the CAR-T cell therapy for the treatment of advanced PCa, particularly for metastatic CRPC (mCRPC), where immune-checkpoint inhibitors with anti-CTLA4 and PD-1 inhibitors, and GVAX and Prost-Vac vaccines showed limited activities [66,67]. Therefore, this innovative approach involves genetically modifying a patient’s T-cells to express a chimeric antigen receptor (CAR) that targets prostate cancer cells. One of the key targets for CAR-T therapy in PCa is the prostate stem cell antigen (PSCA), which is found at higher levels in cancerous cells compared to normal tissues. This specificity helps CAR-T cells to effectively target and kill cancer cells while sparing healthy cells. Clinical trials have provided encouraging results. In a Phase 1 trial, PSCA-directed CAR-T cells demonstrated safety and potential efficacy. Notably, some patients showed significant declines in prostate-specific antigen (PSA) levels and improvements in imaging results. In addition, a subset of patients showed a dynamic change in the activation of peripheral blood endogenous, CAR-T cell subsets, TCR repertoire diversity, and alteration in the TME. However, managing side effects, such as cytokine release syndrome and cystitis, remains a crucial aspect of treatment [68] (Table 2; Figure 1). Finally, another emerging therapeutic strategy is the cancer-associated-fibroblast (CAF). It has been shown that CAFs promote cancer cell survival and proliferation, as well as facilitate metastasis by altering the composition of the tumor extracellular matrix (ECM), and they play a role in developing an immunosuppressive TME [69]. However, recent studies reported the use of CAFs as a treatment option for PCa. One of these therapeutic approaches is to deplete targeting surface molecules of CAFs. Regarding this, fibroblast activation protein (FAP) is expressed on the surface of CAFs in several solid tumors, and its overexpression is correlated with a poor prognosis, and in PCa this protein is associated with PTEN copy number loss. It has been reported that specific sequences designed to silence genes critical for CAF survival or function, such as loading an FAP antibody onto a nanoparticle, inactivated CAFs via a downregulation of CXCL12, inducing a suppression of PCa cells phenotype [70]. Another CAF therapeutic option is to disrupt the signals and interactions that CAFs use to support tumor growth and progression. Recently, the literature demonstrated that inhibition of chemokines/cytokines and growth factors can reduce tumor progression, such as TGFβ1, which is involved in the mechanism of tumor progression and immune evasion. Recently, it has been reported that TGFβ-receptor I antagonist, galunisertib, significantly reduced the tumor growth in a model mouse via regulation of a signaling circuit that involved chemokines like CXCR2 and CXCR5, suggesting that this antagonist could be used as a robust therapeutic target for PCa [71]. Finally, re-inducing activated CAFs to a quiescent state closer to normal fibroblasts (NFs) is indeed a promising therapeutic strategy. This approach aims to reverse the tumor-promoting activities of CAFs, potentially transforming them into cells that support normal tissue homeostasis rather than tumor progression.

New evidence suggests that CAFs are involved in the regulation of tumor metabolism, acting on the dysregulation of various metabolic pathways. Recently, it has been demonstrated that treatment by glucocorticoid induces alteration in the glucocorticoid receptor signaling modifying morphology of CAFs, but also influences proliferation, colony formation, and the 3D-spheroid growth mechanism in several PCa cell models. In this context, it has been observed that there is a clear involvement of glucocorticoid in the regulation of epithelial tumor cell growth, and it is considered an important tool for therapeutic strategies [72]. In conclusion, by targeting CAFs through these various strategies, it is possible to disrupt their supportive role in tumor progression and enhance the effectiveness of cancer treatments (Table 2; Figure 1).

The implementation of radiolabels has revolutionized the landscape of PCa. The theranostic concept involves the use of a single vector to detect the molecular target and to perform therapy. Prostate-specific membrane antigen (PSMA) tracers were initially used as an imaging technique. Afterwards, PSMA labeling with isotopes exhibiting cytotoxic abilities was approved for radioligand immunotherapy in the metastatic stage of PCa, where PMSA or Gastrin-releasing peptide receptor (GRPR) resulted in overexpression [73,74]. The effectiveness of radionuclide therapy arises from its mechanism of action, as a radioactive isotope is linked to a vector molecule that targets tumor cell receptors and/or the tumor microenvironment. Promising radioactive pharmaceuticals are being evaluated in clinical trials, most of which have not been completed yet [75]. Despite the important advancements reached by TRT in the field of personalized therapy, TRT still exhibits some limitations related to efficacy, stability, and side effects [76]. Therefore, further studies are needed before TRT can be used in routine clinical practice, mainly in combination with other treatment modalities and in less advanced forms of PCa (Table 2; Figure 1).

**Table 2:** Preclinical models and new therapeutic strategies for advanced PCa management.

	Preclinical Models	Aims of Tool	Advances	Limitations	References
PDX (Patient-derived xenograft model)	Transplantation of human tumor tissue into an immunodeficiency animal model	More accurate reproduction of tumor’s biology and complexity of TME by human tumor grown in animal model	Useful tool to a personalized therapeutic strategy	Lack of functionality of the human immune system in the host animal; loss of human stromal component in the animal model	[61]
PDOs (Patient-derived organoids model)	3D cultures of patient tissues by a basement membrane (Matrigel)	Development of organoids that reproduce the molecular and clinical diversity of PCa, as various genetic mutations	Mimic of the complexity of TME; a useful tool to test new drugs in PCa-resistant therapeutic strategies	High cost and further protocol standardization	[60,63,64,65]
	**New therapeutic strategies**	**Aims**	**Advances**	**Limitations**	**References**
CAR-T cell therapy	Genetic modification of patient’s T-cells expressing chimeric antigen receptor (CAR) targeting for PCa cells	CAR-T cells target and kill cancer cells	Low levels of PSA and improvement in imaging results; activation of peripheral blood endogenous, TCR repertoire diversity; alteration of TME	Collateral effects such as cytokine release syndrome and cystitis	[68]
Cancer-Associated-Fibroblast (CAF) therapy	Co-culturing of human tumor tissue with CAF	Disruption of tumor progression and enhancement of cancer therapy	Useful tool for new treatment option of PCa; inhibition of chemokines/cytokines and growth factors	Complex interaction within TME due to the heterogeneity and plasticity of CAF	[69,70,71,72]
Targeted Radionuclide therapy (TRT)	Radioactive isotope linked to a vector molecule specifically targeting tumor cell receptors or the tumor microenvironment (PSMA/GRPR)	Improving both overall survival and progression-free survival of patients with mCRPC	A useful tool for personalized treatment of mCRPC; Integration of TRT with other treatment modalities to optimize mCRPC therapy	Radiopharmaceuticals exhibit high tissue absorption, low energy transfer, and a broad radiation spectrum	[73,74,75,76]

## 6. Conclusions

In this review, we aimed to discuss the relationship between PCa and modifiable, lifestyle, and environmental factors and the emerging strategies to optimize the treatment of advanced PCa.

The data emerging from the literature strongly suggest that unhealthy habits and chronic exposure to environmental pollution may have a role in PCa development and progression. These modifiable players interacting with unmodifiable risk factors can lead to the creation of a heterogeneous tumor microenvironment that has a critical role in the progression to a more aggressive tumor and difficult-to-treat stages. The development of preclinical models of PCa is fundamental not only to better understand the molecular mechanisms driving advanced androgen-resistant forms of PCa, but also to identify new therapeutic tools allowing clinicians to adopt personalized therapy. Although the main aim of the research is to find innovative and efficacious new therapeutic approaches in the field of therapy-resistant forms of cancer, public prevention, such as encouraging balanced nutrition and an active lifestyle, remains the gold standard in counteracting the cancer onset and detecting the disease early. On the other hand, the goverments should improve the laws to reduce the environmental exposure because experimental and clinical studies clearly demonstrate that pollutants have a pathogenetic role in the onset of tumors.

## Data Availability

No new data were created or analyzed in this study.
